# Novel Stability-Indicating RP-HPLC Method for the Simultaneous Estimation of Clindamycin Phosphate and Adapalene along with Preservatives in Topical Gel Formulations

**DOI:** 10.3797/scipharm.1404-01

**Published:** 2014-06-20

**Authors:** Prakash B. Modi, Nehal J. Shah

**Affiliations:** ^1^ Analytical Research & Development, IPDO, Dr. Reddy’s Laboratories, Bachupally, Hyderabad-500072, Andhra Pradesh, India.; ^2^ School of Pharmacy, RK University, Kasturbadham, Rajkot-360020, Gujarat, India.; ^3^ Indubhai Patel College of Pharmacy and Research Centre, Dharmaj-388430, Gujarat, India.

**Keywords:** Clindamycin phosphate, Adapalene, Stability indicating, Preservatives

## Abstract

A novel stability-indicating RP-HPLC method was developed for the simultaneous estimation of clindamycin phosphate (hydrophilic), adapalene (hydro-phobic), phenoxyethanol, and methylparaben in topical gel formulations. Optimum chromatographic separation among the analytes and stress-induced degradants peaks was achieved on the XBridge C18 (50 × 4.6 mm, 3.5 µm) column using a mobile phase consisting of a variable mixture of pH 2.50 ammonium hydrogen phosphate buffer, acetonitrile, and tetrahydrofuran with gradient elution. Detection was performed at 210 nm for phenoxyethanol, methylparaben, and clindamycin phosphate and 321 nm for adapalene. The method was optimized with a unique diluent selection for the extraction of clindamycin phosphate and adapalene from the gel matrix. The developed method was validated for method precision, specificity, LOD and LOQ, linearity, accuracy, robustness, and solution stability as per ICH guidelines. The proposed method can be employed for the quantification of clindamycin phosphate, adapalene, phenoxyethanol, and methylparaben in commercial topical gel formulations.

## Introduction

Clindamycin phosphate (CP) is the 2-phosphate ester of clindamycin. Clindamycin (methyl-7-chloro-6,7,8-trideoxy-6-{[(4*R*)-1-methyl-4-propyl-L-prolyl]amino}-1-thio-L-*threo*-α-D-*galacto*-octopyranoside) is a semi-synthetic derivative of lincomycin. It is an antibiotic effective against Gram-positive aerobes as well as Gram-negative and Gram-positive anaerobic pathogens. Topically, it is used for the treatment of acne vulgaris and bacterial vaginosis, which typically leads to the suppression of cutaneous propionibacterium acnes [1–3]. Due to the inhibition of propionibacterium acnes, the free fatty acid level on the skin surface decreases. Clindamycin phosphate applied topically penetrates to a very great extent into open comedones and thus, produces a high percentage of comedones [4].

Adapalene (ADA) is a third generation synthetic retinoid used in the treatment of acne. It is a highly lipophilic compound, derived from napthoic acid, and chemically designated as 6-[3-(1-adamantyl)-4-methoxyphenyl]-2-napthoic acid [5]. Combination therapy with a topical retinoid and an antimicrobial agent, which addresses the majority of the causative factors of acne, is considered a first-line treatment option for almost all patients. Adapalene is also shown to increase follicular penetration of clindamycin [6], and their combination therapy is reported to be highly efficacious and well-tolerated [7, 8].

**Fig. 1.**
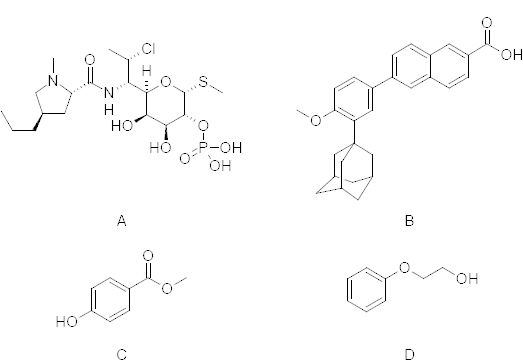
Chemical structures of molecules (A) clindamycin phosphate, (B) adapalene, (C) methylparaben and (D) phenoxyethanol

A preservative system is an integral part of topical formulations to prevent microbial growth during storage. The antimicrobial and antifungal properties make preservatives an integral part of the product formulation [9]. Phenoxyethanol (PHE), methylparaben (MP), and propylparaben are the common preservatives in aqueous-based topical formulations and to establish their effectiveness throughout their shelf life, monitoring of these preservatives is mandatory in pharmaceutical products [10, 11]. Chemical structures of PHE, MP, CP, and ADA are represented in Fig. 1.

Literature surveys of PHE, MP, CP, and ADA revealed that there are numerous analytical methods such as spectrophotometric [12, 13], HPLC [14–23], IVRT [24], and LCMS [25], which are reported either alone or in combination with other components. Combination therapy of retinoid (ADA) and antibacterial agent (CP) is effective against p. acnes and it is available on the market as a topical gel formulation. Individual CP and ADA gel formulations are also available on the market. PHE and MP are the most commonly used preservatives in CP and ADA gel formulations. There are no RP-HPLC methods available for the simultaneous estimation of CP and ADA along with the preservatives. Individual measurement of each component in the topical formulation is a time-consuming process and requires more resources. Hence, there is a need for a faster and more accurate RP-HPLC method for the simultaneous estimation of PHE, MP, CP, and ADA in topical gel formulations.

The aim of the present study was to develop and validate a novel stability-indicating RP-HPLC method for the simultaneous estimation of CP (hydrophilic) and ADA (hydrophobic) along with preservatives (PHE and MP) in topical gel formulations. The method was thoroughly validated for method precision, accuracy, linearity, LOD and LOQ, specificity, solution stability, and robustness as per ICH guidelines.

## Experimental

### Instrumentation

The Waters HPLC system, consisting of a 2695 separation module, 2996 PDA detector, and Empower software for data acquisition, was used for the research. The XS205 Dual Range Balance (Make: Mettler Toledo), Orion 3Star pH meter (Make: Thermo Electron Corporation), ultrasonic bath (Make: Bandelin Sonorex), and Heraeus Biofuge Stratos Centrifuge (Make: Thermo Electron Corporation) were used for the research.

### Standard, Chemicals, and Reagents

PHE (potency: 99.6%), MP (potency: 99.7%), CP (potency: 842.3 µg/mg), and ADA (potency: 99.5%) standards were provided by Dr. Reddy’s Laboratories (Hyderabad, India). Methanol, acetonitrile, 1,4-dioxan, and tetrahydrofuran of HPLC grade (RFCL, India) were used during the study. Chemicals such as ammonium dihydrogen phosphate, potassium dihydrogen phosphate, and phosphoric acid of AR grade (RFCL, India) were used during the study. Purified water was obtained from a Milli-Q system, Millipore (Milford, MA). Syringe filters of 0.45 µm porosity (MDI, India) were used for the study. Topical gels of CP and ADA (Clindapene, Adacin, and Deriva C Gel) were purchased from the market.

### Chromatographic Conditions

Separation of PHE, MP, CP, and ADA was achieved on the XBridge C18 (50 x 4.6 mm, 3.5 µm) as the stationary phase using mobile phase A, which consisted of pH 2.50 ammonium dihydrogen phosphate buffer (5.70 g of ammonium dihydrogen phosphate dissolved in 1000 mL of water and adjusted pH 2.50 with phosphoric acid) and acetonitrile in the ratio of 84:16 (v/v) and mobile phase B, which consisted of acetonitrile and tetrahydrofuran in the ratio of 80:20 (v/v) with gradient mode. Separation was achieved with following gradient program; time (min)/mobile phase A (%)/mobile phase B (%): 0/100/0, 8/100/0, 9/24/76, 13/24/76, 14/100/0 at a flow rate of 1.0 mL/min with a column temperature of 40°C. The injection volume was 5 µL. The chromatograms were recorded at 210 nm for PHE, MP, and CP and at 321 nm for ADA.

### Extracting Solvent (Diluent)

Tetrahydrofuran was followed by the diluent as a mixture of 1,4-dioxane: water (50:50).

### Standard Solution Preparation

About 300 mg of CP was accurately weighed and transferred into a 25-mL volumetric flask, then 15 mL of diluent was added and the flask was sonicated to dissolve it. Then it was made up to volume with diluent (solution A). An accurately weighed amount of about 125 mg of PHE, 50 mg of MP, and 50 mg of ADA were put into a 25-mL volumetric flask, 15 mL of tetrahydrofuran was added, and the mixture was sonicated to dissolve it. Then it was made up to volume with tetrahydrofuran (solution B). Five mL of solution A and 2.50 mL of solution B were transferred into a 50-mL volumetric flask, 7.50 mL of tetrahydrofuran was added, and it was made up to volume with diluent to obtain a solution containing 250 µg/mL, 100 µg/mL, 1000 µg/mL, and 100 µg/mL of PHE, MP, CP, and ADA, respectively.

### Assay of Market Formulations

An accurately weighed amount of about 5000 mg of gel was transferred into a 50-mL volumetric flask, 10 mL of tetrahydrofuran was added and sonicated for 5 minutes with intermittent shaking. Then 25 mL of diluent was added and again sonicated for 10 minutes with intermittent shaking. It was cooled to room temperature and made up to volume with diluent. A portion of the solution was centrifuged at 5000 rpm for 10 minutes, a supernant solution was taken and filtered through a 0.45 µm PVDF membrane filter.

## Results and Discussion

### Method Development and Optimization

Dual wavelengths of 210 nm for the detection of PHE, MP, and CP and 315 nm for ADA were selected based on reasonable response and minimum baseline noise throughout the chromatographic run.

The most important aspect in the LC method development was the achievement of sufficient resolution with acceptable peak symmetry in a reasonable analysis time. To achieve this goal, several experiments were carried out in order to optimize both the stationary and mobile phases. During the optimization of chromatographic conditions, initially, the isocratic method was employed using a mixture of pH 2.50 KH_2_PO_4_ buffer: acetonitrile (775: 225) as the mobile phase, the Hypersil BDS C8 as the stationary phase, and the HPLC equipped with a PDA detector for separation [14]. The PHE peak was separated well, poor separation between the MP and CP peaks was observed, and the ADA peak was not eluted with this isocratic run. Due to the hydrophobic nature of ADA, the mobile phase required a high amount of organic phase to elute the ADA peak. Gradient mode was employed using a variable mixture of pH 2.50 KH_2_PO_4_ buffer and acetonitrile. Simultaneously different stationary phase C8 columns (Hypersil BDS C8, Symmetry C8, and Luna C8) were assessed to enhance the separation between the MP and CP peaks and the early elution of the ADA peak. Poor separation was observed between the MP and CP peaks, precipitation and an HPLC system pressure drop were observed with a variable mixture of the mobile phase containing more than 70% acetonitrile.

**Fig. 2.**
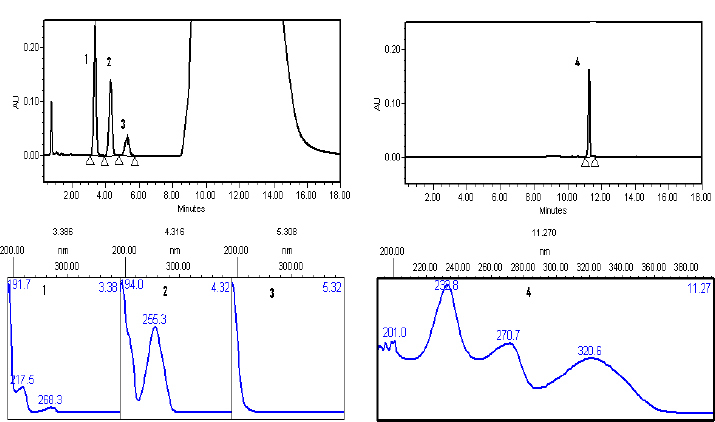
Typical chromatogram of the standard with the UV spectra (1) PHE, (2) MP, (3) CP, and (4) ADA

ADA is soluble in tetrahydrofuran and therefore, we added tetrahydrofuran in the mobile phase to improve the symmetry of the ADA peak. Several mobile phases constituted of a variable mixture of pH 2.50 ammonium dihydrogen phosphate buffer, 0.1% glacial acetic acid, acetonitrile, methanol, and tetrahydrofuran were evaluated along with stationary phases such as the Hypersil BDS C18, XBridge C18, and Inertsil ODS C8 for the sufficient resolution between the MP and CP peaks and also early elution of the ADA peak with a symmetric peak. Successful resolution between the PHE, MP, CP, and ADA peaks with a reasonable run time were achieved by using the XBridge C18 (50 x 4.6 mm, 3.5 µm) as the stationary phase and a variable mixture of mobile phase A (pH 2.50 ammonium dihydrogen phosphate:acetonitrile, 84:16, v/v) and mobile phase B (acetonitrile:tetrahydro-furan, 80:20, v/v) with gradient mode (Fig. 2).

### Diluent Optimization

MP and PHE have very good solubility in methanol, acetonitrile, tetrahydrofuran, and 1,4-dioxane. CP is freely soluble in water, while ADA is freely soluble in tetrahydrofuran.

**Fig. 3.**
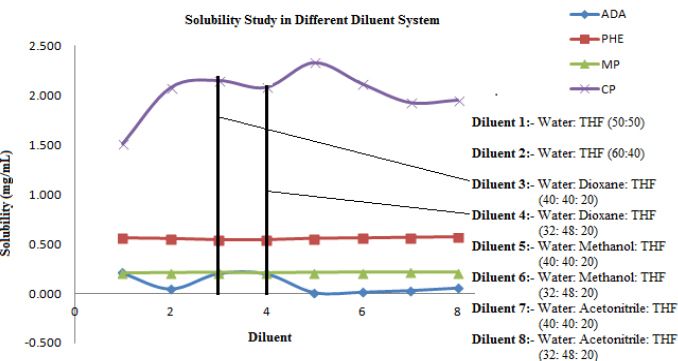
Representative graph of the diluents optimization study

There is a complexity in the diluent selection for the CP and ADA mixture to get the desired solubility of both analytes to achieve the test concentration. The increase in the water concentration of the diluent resulted in the precipitation of ADA, while an increase in tetrahydrofuran resulted in the precipitation of CP and poor peak symmetry of CP was observed. To get the desired solubility of PHE, MP, CP, and ADA as well as the deformation of the gel matrix and extraction of all the analytes from the matrix, different mixtures of water, tetrahydrofuran, acetonitrile, methanol, and 1,4-dioxane were tried. The desired solubility of PHE, MP, CP, and ADA with the desired extraction of all analytes was achieved with a mixture of water:1,4-dioxane:tetrahydrofuran (40:40:20) as diluent (Fig. 3).

### Method Validation

The proposed method for the simultaneous estimation of PHE, MP, CP, and ADA was validated for parameters like specificity, method precision, LOD and LOQ, linearity, accuracy, ruggedness, solution stability, and robustness as per International Conference on Harmonization guidelines [26].

### Method Precision

The intraday and interday precision for the proposed method were performed by the preparation of replicate samples of the same lot of the formulation and analyzed as per the developed method within the day and on a different day. We calculated the % assay of PHE, MP, CP, and ADA in replicate sample preparations. The % RSD of the assay of all the analytes was found to be < 2.0% for the intraday and interday precision (Table 1). This indicates that the method is precise with respect to the reproducibility of the results within the same day or on a different day.

### Specificity

**Fig. 4.**
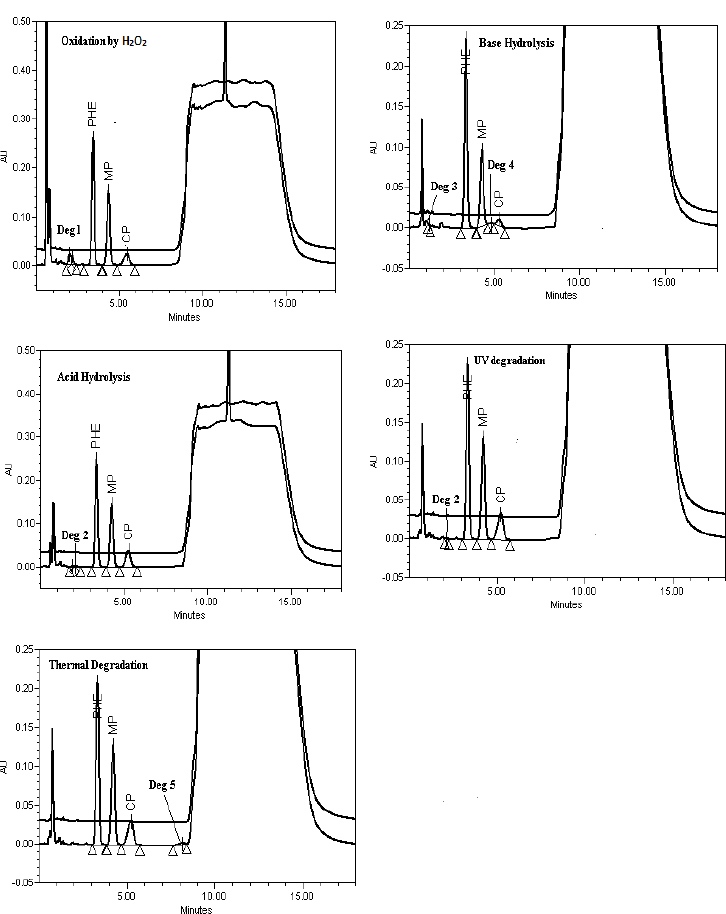
Representative chromatograms of the forced degradation study (210 nm)

Specificity is the ability of the method to access unequivocally the analyte in the presence of the components that may be expected to be present, such as impurities, degradation products, and matrix components. Gel samples were exposed to acid hydrolysis (5 N HCl), base hydrolysis (5 N NaOH), oxidation reaction by H_2_O_2_ (30% H_2_O_2_), photodegradation, and thermal degradation. The stressed samples were injected into the HPLC with a PDA detector as per the developed method and evaluated for the % degradation and peak purity of the PHE, MP, CP, and ADA peaks (Tab. 2). CP was susceptible to acid hydrolysis, base hydrolysis, oxidation, and thermal degradation and hence, the assay of CP had significantly dropped during these conditions. MP was susceptible to base hydrolysis, resulted in a drop in the assay during base hydrolysis. However, PHE and ADA did not show any significant degradation during the forced degradation study. The peak purity of the CP, MP, CP, and ADA peaks were passed in all the stressed samples; this indicates that the method is capable to determine PHE, MP, CP, and ADA in the presence of degradant and excipient peaks (Fig. 4+5), enabling the stability-indicating power of the method.

**Tab. 1.** Results of method precision, LOD, LOQ, and linearity

Parameter	PHE	MP	CP	ADA
Intraday precision				
Mean % Assay (n=6)	97.0	97.2	104.3	100.3
% RSD: NMT 2.0%	1.60	1.60	1.20	0.90
Interday precision				
Mean % Assay (n=6)	96.3	97.1	102.6	101.5
% RSD: NMT 2.0%	1.20	1.10	1.50	1.0
LOD (μg/mL)	0.3	0.2	7.0	0.2
LOQ (μg/mL)	1.0	0.6	20.0	0.5
Linearity	Y = 10249x	Y = 19525x	Y = 698.93x	Y = 12830x
Linearity Equation	+ 1424.8	- 18781	+ 242.94	- 3646
Corr. Coefficient (r)	1.0	0.9989	0.9994	0.9999
Concentration range(μg/mL)	1.0-375.0	0.1-650.0	20.0-1500.0	0.5-150.0

**Tab. 2.** Results of the forced degradation study

Component		Stress condition				
		Acid degrad. 0.3 mL 5 N HCl/70°C/10 min	Base degrad. 0.3 mL 5 N NaOH/70°C/10 min	Oxidation 0.5 mL 30% H_2_O_2_/70°C/10 min	Thermal degrad. 80°C/12 hrs	UV degrad. UV light/
	PA	0.152	0.173	0.274	0.156	0.14
PHE	TH	1.623	1.685	1.357	1.911	1.989
	% Deg	Nil	Nil	Nil	Nil	Nil
	PA	0.116	0.272	0.072	0.128	0.128
MP	TH	1.436	1.557	1.122	1.713	1.782
	% Deg	Nil	25.0%	Nil	Nil	Nil
	PA	0.927	1.877	3.293	0.844	0.677
CP	TH	3.564	12.174	4.243	4.659	4.81
	% Deg	2.20%	41.00%	16.60%	1.90%	1.40%
	PA	0.218	0.106	0.154	0.37	0.258
ADA	TH	1.157	1.171	1.045	1.252	1.269
	% Deg	Nil	Nil	Nil	Nil	Nil

PA…purity angle; TH…threshold angle; peak purity criteria: PA < TH.

**Fig. 5.**
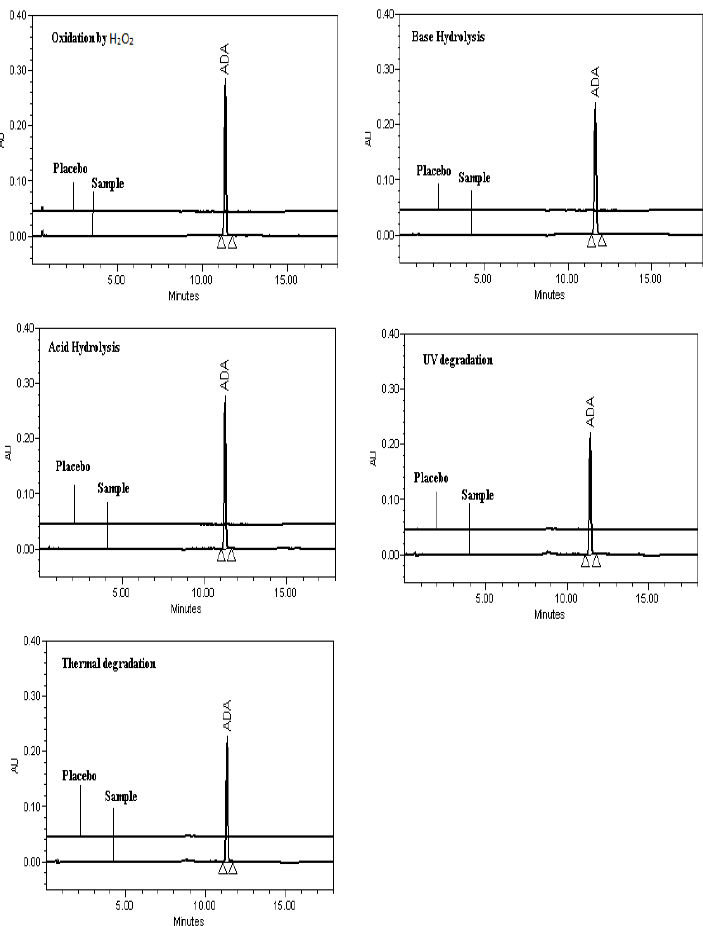
Representative chromatograms of the forced degradation study (321 nm)

### Limit of Detection (LOD) and Limit of Quantification (LOQ)

**Fig. 6.**
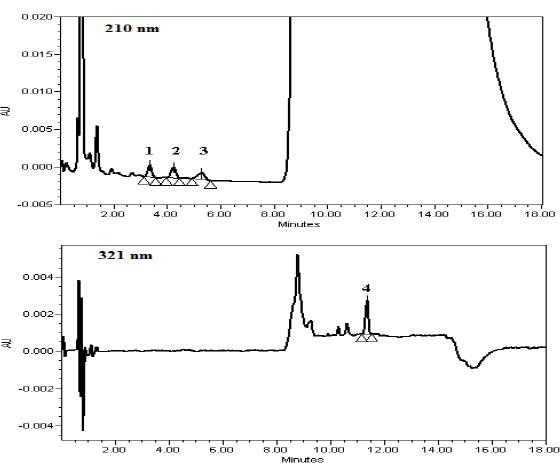
LOD and LOQ chromatograms containing (1) PHE, (2) MP, (3) CP, and (4) ADA

The LOD is the lowest amount of analyte in a sample which can be detected, but not necessarily quantitated as an exact value, while the LOQ is the lowest amount of analyte in a sample which can be quantitatively determined with suitable precision and accuracy. The LOD and LOQ for PHE, MP, CP, and ADA were determined as per ICH guidelines (Fig. 6). The LOD and LOQ values for PHE, MP, CP, and ADA are presented in Table 1.

### Linearity

The linearity of the proposed HPLC method was evaluated by analyzing a series of solutions containing PHE, MP, CP, and ADA in the concentration range of the LOQ to 150% of the target assay concentration. There are the plotted graphs of the concentration in µg/mL versus area for PHE, MP, CP, and ADA. The correlation coefficients of the linearity plots were found to be more than 0.99 for all the analytes, enabling the linear behavior of the method in the established concentration range. PHE, MP, CP, and ADA showed linearity in the range of 1.0–375.0 µg/mL, 0.6–150.0 µg/mL, 20.0–1500.0 µg/mL, and 0.5–150.0 µg/mL, respectively. Linear regression equations and correlation coefficient are presented in Table 1.

### Accuracy

The accuracy of the proposed method was evaluated by varying the sample weight from 50% to 150% of the test concentration. We analyzed the recovery samples as per the proposed method. We measured the recovered concentration versus the added concentration for PHE, MP, CP, and ADA and calculated the % recovery at all levels. The % recovery for all the analytes was found to be more than 97.0% (Table 3); this indicates the accuracy of the proposed method.

**Tab. 3.** Results of the recovery study

Spiked Level	Component	Amount Added(mg/mL)	Amount Recovered(mg/mL)	% Recovery	% RSD
	PHE	1.21	1.20	98.8	1.72
50% (n=3)	MP	0.486	0.494	101.7	0.91
	CP	5.22	5.16	99.0	1.67
	ADA	0.50	0.51	101.1	0.49
	PHE	2.43	2.42	99.9	2.27
100% (n=3)	MP	0.97	0.97	100.1	2.15
	CP	10.43	10.51	100.7	1.01
	ADA	1.00	1.01	100.5	0.48
	PHE	3.64	3.60	99.1	0.17
150% (n=3)	MP	1.46	1.44	98.5	0.88
	CP	15.65	15.49	99.0	1.75
	ADA	1.50	1.54	102.3	0.79

**Tab. 4.** Results of the robustness study

Component		Control condit.	Flow (mL/min)		Col. Temp		Buffer pH		Mobile Phase A (v/v)	
			0.9	1.1	35°C	45°C	2.30	2.70	Buff:ACN 82:18	Buff:ACN 85:15
	T	1.0	1.0	1.1	1.1	1.1	1.1	1.1	0.9	1.1
PHE	TP	2631	2935	2812	2706	2544	2877	2911	3156	2755
	%RSD	1.75	1.25	0.98	1.3	1.19	0.94	1.39	1.12	1.19
	RT	3.39	3.67	3.08	3.48	3.27	3.38	3.33	3.85	3.26
	T	0.9	1	1.1	1.1	1.1	1.1	1.1	0.9	1.1
MP	TP	2747	2833	2678	2589	2390	2636	2670	2980	2577
	%RSD	1.78	1.32	0.75	1.03	1.36	1.44	1.2	0.78	1.55
	RT	4.32	4.67	3.93	4.53	4.02	4.31	4.24	4.99	4.13
	T	0.9	0.9	1	0.9	1.1	0.9	0.9	0.8	1
CP	TP	1947	2057	2004	2132	1723	1927	1855	1895	1730
	%RSD	1.61	1.56	1.13	0.76	0.81	1.1	1.29	1.04	1.68
	RT	5.31	5.7	4.87	5.5	5.27	5.41	5.1	7.58	4.95
	T	1.0	1.1	1.1	1.2	1.1	1.1	1.1	1	1
	TP	78698	80123	75776	77336	75349	74568	77326	82058	71970
ADA	%RSD	1.58	1.03	0.87	1.09	1.1	1.25	0.97	1.37	1.43
	RT	11.27	11.86	11.14	11.42	11.29	11.27	11.23	11.29	11.14

TP…theoretical plate count; %RSD (n=5): NMT 2.0%; RT…retention time.

### Solution Stability

The stability of the standard solution as well as the sample solutions was examined at room temperature. No degradation (< 2.0%) of PHE, MP, CP, and ADA was observed in the standard and sample solution within 24 hours at room temperature. This indicated that the standard and sample solution were stable for 24 hours at room temperature.

### Robustness

Robustness of the method was examined by evaluating the influence of small variations in different conditions such as flow rate (0.9–1.1 mL/min), column temperature (35–45°C), buffer pH (2.30–2.70), and mobile phase A composition (buffer: acetonitrile, 82:18–85:15, v/v). The standard solution was injected with each of the variable chromatographic conditions and evaluated for system suitability compliance. Results are summarized in Table 4 and enable the proposed method to be robust with respect to changes in the above variable chromatographic conditions.

### Analysis of Market Formulations by the Proposed Method

Three different marketed gel formulations containing CP and ADA were analyzed in triplicate as per the proposed method and we calculated the % assay of CP, ADA, and preservatives in the sample preparation. The results are summarized in Table 5.

**Tab. 5.** Assay results of the market formulations

Market formulation	Component			
	PHE	MP	CP	ADA
Clindapene Gel	96.3 ± 1.29	97.0 ± 1.76	104.8 ± 1.41	100.2 ± 1.47
Adacin Gel	-	-	97.2 ± 1.49	103.0 ± 0.69
Deriva C Gel	-	-	98.4 ± 1.33	104.4 ± 0.40

%Assay ± RSD (n=3).

## Conclusion

The proposed HPLC method is novel, sensitive, and accurate for the simultaneous estimation of PHE, MP, CP, and ADA in topical gel formulations. This method shows the separation of hydrophilic (CP) and hydrophobic (ADA), along with preservatives in topical gel formulations. This method describes the dual wavelengths: 210 nm for PHE, MP, CP and 315 nm for ADA detection. This method enables a complex diluent selection study to get the desired recovery of PHE, MP, CP, and ADA from the gel matrix. The method demonstrated the optimum separation of all the analytes in the presence of degradant and excipient peaks of the formulations in all stressed samples, enabling the stability-indicating power of the method. The proposed method was validated for method precision, linearity, LOD and LOQ, accuracy, robustness, and solution stability as per ICH guidelines. The proposed method can be employed for the quantification of PHE, MP, CP, and ADA in commercial topical gel formulations.
